# Positive Pressure for Obesity Hypoventilation Syndrome

**DOI:** 10.1155/2012/568690

**Published:** 2012-10-11

**Authors:** Arijit Chanda, Jeff S. Kwon, Armand John Wolff, Constantine A. Manthous

**Affiliations:** ^1^Section of Pulmonary, Critical Care, and Sleep Medicine, Bridgeport Hospital, Bridgeport, CT 06610, USA; ^2^Hospital of Central Connecticut, 100 Grand Street, New Britain, CT 06050, USA

## Abstract

Obesity is increasing world-wide; obesity hypoventilation syndrome (OHS), formerly Pickwickian syndrome, has increased in parallel. Despite its prevalence, OHS has not been studied well, but there is abundant evidence that it is tightly linked with sleep-disordered breathing, most commonly obstructive sleep apnea. This article reviews the pathophysiology of OHS as well as the literature regarding the benefits of treating this disorder with positive airway pressure. We also emphasize that while positive pressure treatments may temporize cardiopulmonary disease progression, simultaneous pursuit of weight reduction is central to long-term management of this condition.

## 1. Introduction

In their extraordinary paper of 1956, Burwell and colleagues introduced and carefully catalogued clinical and physiologic abnormalities of a syndrome of “extreme obesity associated with alveolar hypoventilation [1].” Referencing a description of Mr. Wardle's servant, Joe, in Charles Dicken's *The Posthumous Papers of the Pickwick Club*, this group coined the term “Pickwickian Syndrome” to describe patients with marked obesity, somnolence, twitching, cyanosis, periodic respiration, polycythemia, and right ventricular hypertrophy and failure. The first case series of 17 patients, reported in 1959 by Hackney and colleagues, demonstrated restrictive respiratory physiology and attenuated response to hypercapnia in the cohort [2]. The eponym–Pickwickian Syndrome–describes the most severe subtype of this disorder and is used less frequently in the 21st century, in favor of the more inclusive obesity hypoventilation syndrome (OHS) defined as chronic hypercapnia (awake *P*CO_2_ > 45 mmHg at sea level), hypoxia (*P*O_2_ < 70 mmHg), and sleep-disordered breathing in obese (body mass index, BMI > 30 kg/m^2^) patients without other causes of alveolar hypoventilation [3, 4]. As obesity has become epidemic in the United States [5, 6] and worldwide [7], OHS has emerged as a relatively common cause of chronic hypercapnic respiratory failure [8].

## 2. Pathophysiology

The pathophysiology of OHS is not completely understood [1–4] and may reflect phenotypic complexity and admixture with various other respiratory diatheses (e.g., tobacco-related, obstructive lung disease). Several inter-related factors likely contribute to varying degrees in each patient with OHS. It is indisputable that obesity imparts increased mechanical load in the form of decreased chest wall compliance [9].

Accordingly, most patients with OHS demonstrate a restrictive defect on pulmonary function testing, with proportional reductions in both forced expiratory volume in one second (FEV1) and forced vital capacity (FVC) [10, 11]. Expiratory reserve volume (ERV) is often significantly decreased [11]. As BMI increases (e.g., >35 kg/m^2^), functional residual capacity may approach residual volume (RV) or closing volume (CV) causing atelectasis—especially in dependent lung regions—and hypoxemia. A study by Steier and colleagues demonstrated that compared to non-obese controls, obese individuals have increased work of breathing that requires an increase in neural respiratory drive. This was exacerbated when subjects were in a supine posture compared to sitting. This study also showed that obese subjects developed intrinsic positive end-expiratory pressure (PEEP) while supine [12]. It stands to reason that reduced chest wall compliance and these other physiologic abnormalities reduce respiratory reserve (e.g., ability to respond to exercise or other ventilatory challenges), but it has not been shown conclusively that chronic hypercapnic respiratory failure results from neuromuscular capacity being outstripped by demand. Chest wall mechanics alone do not account for chronic hypercapnic respiratory failure, because continuous positive airway pressure (CPAP)—titrated to eliminate obstructive apneas—can normalize *P*CO_2_ in some patients (vide infra).

It has been long recognized that obese individuals have greater CO_2_ production compared to nonobese individuals, along with increased consumption of oxygen [13, 14]. Although increased CO_2_ production due to obesity alone is unlikely to result in hypercapnia, it is reasonable to assume it plays a contributing role. Other factors may include the abnormal central ventilatory drive that has been demonstrated in many eucapnic [15] and hypercapnic [16] obese patients. Reduced responsiveness to hypercapnia appears to be a critical and common feature in the pathogenesis of OHS [3, 4, 11, 15]. Leptin levels may play a mediating role. A hormone produced by adipocytes, leptin acts on the hypothalamus to reduce appetite and increase energy expenditure. It also stimulates ventilation [17]. Either a deficiency of leptin or decrease in its activity (e.g., leptin resistance) could contribute to hypoventilation even in the absence of excessive mechanical loading [18–20]. Interestingly, leptin levels rise when OHS-related hypercapnia is reversed with positive pressure ventilation (PPV) [18].

Finally, sleep-disordered breathing, especially obstructive sleep apnea (OSA), may impact ventilatory drive and/or contribute directly to chronic hypercapnia. OSA is the most common sleep disorder and is present in more than 90% of patients with OHS [21]. A minority of patients with OHS have nonobstructive sleep-disordered breathing. A novel hypothesis has been offered by Berger and colleagues to explain incremental hypercapnia related to sleep-disordered breathing in patients with OHS [22]. These authors suggest that intermittent periods of CO_2_ loading during apnea/hypopnea may cause gradual, compensatory, increases in bicarbonate buffering during sleep. *P*CO_2_ undulates up and down during sleep-disordered breathing: if the inter-apneic period shortens or the duration of apneas increases, CO_2_ may accumulate during apneic/hypopneic phases. If arousal and resumption of sleep occurs before eucapnia returns, residual hypercapnia may provide a driving force for renal compensation (i.e., reclamation of bicarbonate). And if during the day, when obstructions no longer occur, there is insufficient time to excrete the retained bicarbonate load, patients would enter the next sleep cycle with a subtle metabolic alkalosis that would worsen with time. If true, this idea, that during sleep bicarbonate buffering impacts the awake CO_2_ set point might have implications for management in the future. However, there is a minority of patients with OHS who have nonobstructive sleep-disordered breathing [23, 24]. In these patients, the hypothesis put forth by Berger and colleagues would not apply suggesting that hypercapnia in obesity may be a heterogeneous disorder.

## 3. Clinical Features

The diagnosis of OHS is frequently delayed, and most patients are diagnosed in their 6th and 7th decades. The combination of obesity and chronic respiratory acidosis brings them to clinical attention, but most have clinical features that can be helpful in earlier detection and management (i.e., before right heart failure and recurrent bouts of acute on chronic hypercapnic respiratory failure; ACHRF). Most patients with OHS snore, have witnessed apneas, choking episodes while sleeping, excessive daytime sleepiness and morning headaches, very similar to patients with OSA alone [2, 3]. They frequently experience episodes of dyspnea and have a low exercise tolerance which may accompany signs of right heart failure. Excessive daytime sleepiness can result in economic, social, and emotional problems [3, 4]. When tested for neurocognitive performance, patients with OHS often have lower scores compared to obese patients without OHS [8]. Beyond obesity, a large neck circumference, “crowded oropharynx” and abnormally low oxygen saturations on room air are common as BMI increases. A prominent pulmonic component of the second heart sound marks increased right-sided pressures. Lower extremity edema is suggestive of cor pulmonale and connotes advancing right heart failure in the latter phases of OHS [3, 25].

## 4. Morbidity and Mortality

The presence of OHS is associated with an increase in health care utilization, increased medical morbidity, and poorer health outcomes. Patients with OHS experience greater hypersomnia as indicated by a higher Epworth score and have a lower level of social functioning, when compared to the patients with OSA alone [26]. Compared to obese control subjects, patients with OHS are more likely to be diagnosed with congestive heart failure, angina pectoris, cor pulmonale [21], and are more likely to be hospitalized [27]. The number of days spent in the ICU is also significantly higher when compared with matched controls [27] as acute on chronic hypercapnic respiratory failure is a common manifestation of OHS. Patients with OHS are particularly vulnerable to iatrogenic insults (e.g., hyperoxia-induced hypercapnia [28] and narcotic-related complications) and to common causes of acute on chronic respiratory failure (e.g., pneumonia, bronchospasm, congestive heart failure, infections and pulmonary embolus) [29]. Nowbar and colleagues reported that OHS patients required more intensive care unit management, had longer lengths of stay and were more likely to be discharged to a long-term care facility. Further, if left untreated, they had higher mortality compared to matched obese controls [8]. Perez De Llano and colleagues reported that a substantial number of patients with OHS died in a follow-up period of 50 months if they were not assisted with long-term positive airway pressure [30]. Budweiser and colleagues followed 126 OHS patients with a mean BMI of 44.6 kg/m^2^ and *P*CO_2_ of 55 mmHg for a mean of 41 months [31]. All-cause mortality was 12.7% despite 95% adherence to positive pressure therapies and 30% had died by 5 years.

### 4.1. Positive Pressure For OHS

In theory, positive pressure could provide salutatory effects in patients with OHS during inhalation and exhalation. Most OHS patients have OSA, and this certainly contributes to symptoms if not the pathogenesis of chronic hypercapnia (*vide supra*). Reduction or elimination of sleep-related obstructions will provide all of the benefits that have been well documented in patients with simple OSA [32]. To the extent that OSA contributes to very gradual, incremental elevations of bicarbonate promoting chronic hypercapnia [22], continuous positive airway pressure (CPAP) during sleep may also be instrumental in reversing both the symptoms and acid base disturbances of OHS.

Positive pressure ventilation (PPV) provides support of inspiration (i.e., ventilation) usually in addition to providing expiratory positive airway pressure (EPAP) to prevent expiratory collapse of large airways. As outlined above, obesity loads the respiratory muscles during inspiration, eroding respiratory reserve. To the extent that inspiratory loading contributes to the propensity to develop hypercapnia in OHS patients, unloading during sleep [33] may attenuate respiratory muscle fatigue (not with standing that fatigue has not been demonstrated in these patients).

Finally, PPV could be beneficial in OHS patients who develop acute loading that promotes acute on chronic hypercapnic respiratory failure. In this situation, PPV would be used as a “bridge” during sleep and wakefulness until the acute load is reduced sufficiently such that unassisted ventilation is adequate to meet needs. Acute hypercapnia may occur in OHS patients independent of loading, for example, in the subset who retain CO_2_ as a result of excessive oxygen administration or narcotics. In these situations, PPV could diminish both the OSA that inevitably accompanies this form of acute on chronic hypercapnia and unload inspiration [33]. PPV might also provide mandatory backup ventilation until normal ventilatory drive returns.

As an overview for this section, it is important to note that drawing definitive conclusions about precisely how to apply positive pressure is complicated substantially by the fact that clinical studies of patients with OHS are relatively small, heterogeneous (i.e., varying degrees of obesity and sleep disorders), and the precise positive pressure interventions vary or are not described. Nonetheless, some reasonable conclusions can be drawn from the available literature. Additionally, it is also worth emphasizing the obvious: positive pressure is not definitive therapy but rather can help manage, acutely or chronically, acid base disorders and some of the symptoms and long-term consequences (e.g., cor pulmonale) that are inevitable in untreated OHS. While positive pressure may temporize, attention should focus simultaneously on weight loss as underlying primary therapy of these disorders.

## 5. Continuous Positive Airway Pressure (CPAP)

Sullivan and colleagues were the first to apply nocturnal nasal CPAP to 2 patients with OHS (*P*CO_2_ 63 and 55 mmHg). Symptoms (mental dysfunction and right heart failure) improved in the first 3 days. After 23 and 35 days of therapy, the patients slept without airway occlusions or deep oxygen desaturations (to <80%) [34]. In another elegant report, Rapoport and colleagues reported the case of a 52-year-old obese man with “incapacitating daytime hypersomnolence and loud snoring” [35]. At 114 kg, he had *P*CO_2_ of 47–55 mmHg and *P*O_2_ of 55–60 mmHg breathing room air. A polysomnogram revealed obstructive sleep apnea (60 obstructive apneas/h accompanied by desaturations to <75%). The patient refused tracheostomy; the authors applied Sullivan's findings and constructed a nasal CPAP apparatus. CPAP of 10–12 cm H_2_O “obliterated” apneas and eliminated both overt and electroencephalographic evidence of arousals. The physiologic benefits were preserved at 3 weeks, when arterial blood gas had normalized (pH = 7.40, *P*CO_2_ = 40 mmHg) at 9 months. This was the first report of successful, long-term, ambulatory management of OHS with positive airway pressure.

A number of reports have since demonstrated that nocturnal CPAP can reverse daytime hypercapnia and some symptoms of OHS [36–38]. CPAP has also been shown to offset intrinsic PEEP (positive end expiratory pressure) that develops in obese individuals [12]. Banerjee and colleagues compared physiology and sleep studies of 23 severely obese (BMI ≥ 50 kg/m^2^) patients, with moderate-to-severe OSA-only (apnea-hypopnea index ≥ 15/h) to 23 with both OSA and OHS [39]. One night of CPAP titration increased the proportion of rapid eye movement sleep, decreased arousal indexes, and improved nocturnal oxygen desaturations in both groups. Despite similar alleviation of obstructions with CPAP, 43% of the OHS group spent >20% of sleep time with O_2_ saturation < 90% compared to only 9% of the OSA-alone group.

While the long-term efficacy was not reported, this study suggests that in OHS, CPAP alone is not sufficient to treat the sleep-related hypoxemia. The residual oxygen desaturations might reflect atelectasis not alleviated by CPAP or otherwise altered ventilatory physiology. Interestingly, there has been no large prospective study, using a single modus operandi of titration, comparing outcomes of patients with OHS treated with CPAP versus PPV. Indeed, even risks of CPAP failure—20% in one retrospective study [36] have not been well identified [40].

CPAP has not been studied for management of patients with ACHRF where short-term unloading with noninvasive positive airway pressure might be necessary to augment respiratory muscle capacity until loads are reduced. Nor has CPAP been examined for treatment of iatrogenic hypercapnia (e.g., hyperoxia-or narcotic-induced). In a series of 6 stuporous patients (BMI = 52 kg/m^2^) with OHS and acute on chronic hypercapnia (not necessarily respiratory failure), a carefully monitored trial of incremental CPAP and oxygen (titrated to oxygen saturation > 85%), pH increased from 7.23 to 7.35 and *P*CO_2_ decreased from 80 to 64 mmHg after 24 hours of CPAP (7.5–10 cm H_2_O) [41]. All 6 patients regained alertness. While 4 of the 6 were hypoxemic (*P*O_2_ < 60 mm Hg) ruling out hyperoxia-induced hypercapnia, the low basal respiratory rate coupled with stupor raises the question of narcotic-related hypoventilation (not mentioned).

The role of oxygen therapy during positive pressure therapies warrants comment. It is imperative during application of both CPAP and PPV for chronic and ACHRF that care is taken to avoid hyperoxia which can promote hypercapnia in some patients [28] with both acute and chronic respiratory failure. For example, if patients require oxygen initially and slowly improve (e.g., losing weight, improved periodic respirations, etc.), it is theoretically possible that excessive oxygen administration could potentiate chronic hypercapnia. The converse is also true; mucus plugging or intermittent obesity-related atelectasis can cause transient unrecognized nocturnal hypoxemia. To the extent that these patients are “moving targets,” periodic nocturnal pulse oximetry may be helpful to detect overadministration of oxygen unto iatrogenic hypercapnia, and underadministration that might potentiate cor pulmonale and right heart failure.

### 5.1. Positive Pressure Ventilation (PPV) for OHS

Despite the initial early successes with CPAP [31, 32], there is far more data on the efficacy of PPV for OHS. It is not clear why, but likely relates to the fact that many patients with OHS continue to hypoventilate/desaturate even after CPAP has treated obstructions, so during sleep studies such patients are often advanced to PPV, most usually bilevel PAP [36]. There are no large prospective randomized studies comparing CPAP and bi-level PAP for OHS. Piper and colleagues randomized 36 ambulatory and stable patients with OHS, with a mean BMI of 53 kg/m^2^ and baseline *P*CO_2_ of 50 mmHg to CPAP or bi-level PAP in spontaneous mode [37]. After 3 months, mean *P*CO_2_ decreased to a similar degree in both groups (−5.8 versus −6.9 in CPAP versus bi-level PAP resp.), daytime sleepiness had decreased similarly but the Pittsburgh Sleep Quality Index was significantly improved in the bi-level PAP group (both relative to baseline and to the CPAP group). Psychomotor vigilance also tended to be improved in bi-level PAP-treated patients.

A number of other studies have examined physiologic responsiveness of OHS patients to bi-level PAP. Nearly all demonstrate reductions in *P*CO_2_ [18, 30, 36, 37, 43–49], and some showed improved sleep architecture [37, 49] and/or improved symptoms [41, 43] (see Table 1). Mokhlesi and colleagues demonstrated an association of “dose” (i.e., hours of positive pressure each day) with improvements in gas exchange [36]. On average, *P*CO_2_ dropped by nearly 2 mmHg for each hour of use, and near-maximal response was reached with 7 hours of daily adherence. Note again, however, that studies offer variable (often sparse) details on perseverance in mask fitting, patient compliance, and titration of PPV; this methodologic heterogeneity hinders valid meta-analysis of these data. The precise monitoring/techniques applied to avoid hyperoxia-induced hypercapnia in patients receiving concomitant oxygen were also not well described. Furthermore, determining optimal PPV settings for OHS may be hindered by the lack of standardization of scoring respiratory events on PPV. Gonzalez Bermejo and colleagues reviewed polysomnography and polygraph tracings over a 2-year period and published recommendations on how to define and recognize respiratory events on noninvasive ventilation [50]. These events included mask leak, changes in ventilatory drive, upper airway collapse, and patient-ventilator desynchrony. Detailed descriptions and examples were provided in the paper, and the impact of these events on oxygenation, ventilation, and arousals were discussed [50]. Using these guidelines in future studies to standardize scoring of respiratory events on PPV may help to clarify the impact of PPV on OHS patients and determine how to achieve optimal ventilator settings.

The impact of different bilevel PAP modes on normalizing sleep-disordered breathing in OHS patients requires special mention. Bi-level PAP can be set in spontaneous/timed (ST) mode, where a backup respiratory rate with an inspiratory time is used to ensure a certain predetermined level of ventilation. For example, if a patient was placed on bi-level PAP ST with a backup rate of 12 breaths per minute (each respiratory interval = 5 seconds) with a set inspiratory time of 1.0 second, the bi-level PAP would initiate and deliver the IPAP for 1.0 second at least every 5 seconds if the patient failed to spontaneously trigger a breath within that time frame. This is in contrast to bi-level PAP in spontaneous mode (S), where delivery of IPAP is triggered by the patient every breath. Therefore, if the patient on bi-level PAP-S fails to trigger the ventilator, the bi-level PAP would deliver only the EPAP and no back up ventilation would be provided. Theoretically, bi-level PAP-ST can increase minute ventilation compared to bi-level PAP-S by simply increasing the back up respiratory rate and inspiratory time above the patient's spontaneous respiratory rate. However, this may come at the expense of patient-ventilator synchrony and comfort.

Whether bi-level PAP-ST offers a higher level of control of sleep-disordered breathing over bi-level PAP-S in OHS patients was studied by Contal and colleagues. Ten OHS patients underwent 3 separate sleep studies on 3 separate nights, each night on a different bi-level PAP setting. The 3 settings were bi-level PAP-S, bi-level PAP-ST with a low back up rate, and bi-level PAP-ST with a high back up rate. Their study demonstrated that patients on bi-level PAP-ST ventilation had significantly lower obstructive and central apnea indexes and improved oxygenation compared to patients on bi-level PAP-S ventilation [51]. Interestingly, transcutaneous *P*CO_2_ did not differ between modes. Of note, subjects reported greater levels of discomfort with bilevel PAP-ST with a high back up rate compared to bi-level PAP-ST with a lower back up rate suggesting possible patient-ventilator desynchrony [51].

Finally, in the acute care hospital setting, bi-level PAP has been advocated for ACHRF in patients with OHS [52]. Although it has been in our experience that it is used frequently for this situation, there are very few data to demonstrate safety or efficacy in such patients. In a retrospective study that included 22 OHS patients with ACHRF, Perez De Llano and colleagues showed that some patients can be safely treated with bi-level PAP [30]. Gas exchange improved (initial pH 7.28 to treated 7.38; initial *P*CO_2_ 70.6 mmHg to 51.5 mmHg by hospital discharge); but the specific PPV parameters and details of the group were not presented in the paper.

### 5.2. Other PPV

Recently, average volume-assured pressure support (AVAPS) has been trialed for stable OHS patients. AVAPS allows varying pressure support to achieve a preset target tidal volume and may have certain advantages over standard bi-level PAP. During sleep, central respiratory drive and ventilation change depending on sleep stage (REM versus non-REM sleep) and body posture (i.e., supine versus lateral sleep position). Theoretically, AVAPS ensures a more consistent tidal volume and minute ventilation over fixed-level pressure support. Storre and colleagues performed a randomized crossover trial of 10 OHS patients with a mean BMI of 42 kg/m^2^, to bi-level PAP or bi-level PAP with AVAPS [47]. Transcutaneous CO_2_ measurements were significantly better on bi-level PAP with AVAPS but this did not translate to improved quality of sleep or daytime function. A second study reported similar results in *P*CO_2_ (better with bi-level PAP-AVAPS) but at the expense of subjective (relative) discomfort [53]. A recent study prospectively randomized 25 patients with mean BMI=52 kg/m^2^ to bi-level PAP and 25 with mean BMI = 50 kg/m^2^ to bi-level PAP-AVAPS. Hypercapnia improved in both groups to a similar degree (53 to 48 mmHg bi-level PAP; 51 to 47 mmHg bi-level PAP-AVAPS) after 3 months of therapy [48]. Subjects in both groups experienced a similar reduction in daytime *P*CO_2_, Epworth scores, and improved quality of life at 3 months regardless of ventilation mode [48]. Of note in this study is the meticulous protocolized titration of bi-level ventilation, to gain satisfactory control of nocturnal hypoventilation and abolition of obstructive events. As a result, both the AVAPS and fixed pressure groups had a similar back up rate and tidal volume targets [48]. The IPAP and EPAP in both groups were also very similar; this may help to explain the lack of difference between groups [48]. However, it is not known whether AVAPS offers advantages over bi-level PAP in spontaneous mode or in settings where less rigorous titration protocols are available. The potential advantages of AVAPS over fixed level pressure support in improving sleep quality, daytime functioning, and daytime gas exchange in patients with OHS remain controversial.

### 5.3. Additional Considerations for ACHRF in OHS Patients

Patients with OHS and ACHRF require care in a critical care or intermediate care unit until the acute component of their respiratory failure is successfully treated and they can ventilate and oxygenate without mechanical assistance [54]. The acute load that has precipitated ACHRF should be identified and treated. The “usual” suspects include bronchospasm, infections, left heart failure, thromboembolic disease, and myocardial ischemia [29, 54]. Such patients are particularly at risk because, if their acute diathesis worsens before it improves, they may require endotracheal intubation and PPV. Also a subset may be oxygen sensitive [28], so that excessive administration of supplemental oxygen might cause acute on chronic hypercapnia leading to lethargy and need for intubation. Whether to apply nasal or full-face mask positive pressure is a matter of patients' comfort and empiric results. Note that great attention should be paid to mask fitting and starting with lower pressures (allowing patients to acclimate before increasing to higher pressures), lest they develop an aversion and subsequent resistance to positive pressure therapies [42, 55].

Note also those routine supportive therapies, including elevation of the head of the bed, aspiration precautions, nasal bridge or forehead pressure sore precautions, thromboprophylaxis, spontaneous breathing trials every 12–24 hours until liberated, and attention to nutrition, should be implemented to reduce complications of critical illness.

Finally, for patients with substantial cor pulmonale and peripheral edema due to OHS, great care must be taken to manage preload. In the earliest phases of management, when patients may have developed hypoxic pulmonary vasoconstriction [56], aggressive diuresis may not be successful and may actually be harmful, as patients are likely to become volume depleted or even hypotensive if they are over-diuresed. But within 12–48 hours, as hypoxic pulmonary vasoconstriction abates, diuresis is often more successful and if clinicians do not “keep up” with increasing venous return, pulmonary edema might develop [57]. Left heart failure can be difficult to diagnose in patients with chest radiographs that are often already confounded by small lung volumes and overlying adipose tissue. How much to diurese is very much a matter of empiric therapy, taking pains not to “overdo it” in the first 24 hours, and realizing that some patients can be successfully diuresed in excess of 1 liter each day after 24 hours of sufficient oxygenation.

## 6. Conclusions

**Figure 1:**
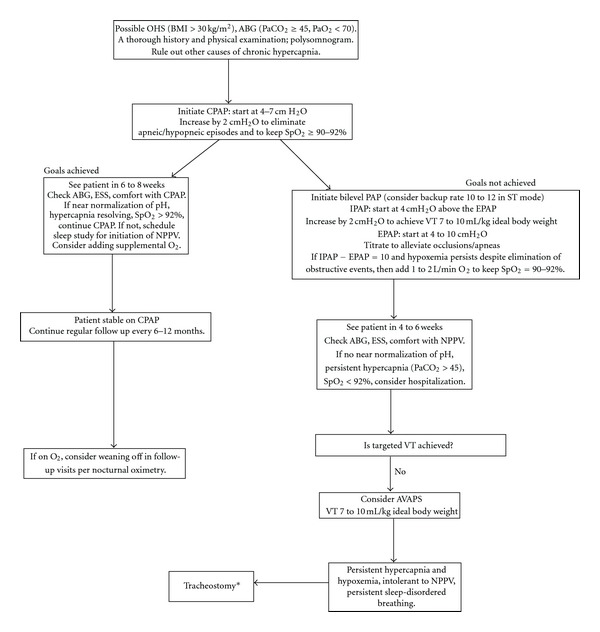
Our sleep lab's approach to patients with OHS. *As with positive pressure therapies, tracheostomy is not a cure—but rather a temporizing measure to reduce propensity for progressive cardiorespiratory failure until weight loss can be achieved. A consensus approach is presented in [42].

CPAP is appropriate first-line therapy for ambulatory OHS patients with stable chronic hypercapnic respiratory failure. If a trial of nocturnal CPAP titration fails to eliminate substantial oxygen desaturations (e.g., <88% for more than a few minutes each night)—as in roughly 20% of patients [36]-either addition of low-dose oxygen or a bi-level PAP trial are indicated even though the single small study comparing CPAP with bi-level PAP did not demonstrate long-term differences in patient outcomes (see Figure 1). Bi-level PAP should begin with EPAP = 4–10 cm H_2_O and pressure support of 4 cm H_2_O. Increases of one or both pressures in increments of 2-3 cm H_2_O until desaturations are eliminated, pressure support >10 cm H_2_O, or patient develops intolerance to therapy. If desaturations persist despite CPAP or bi-level PAP (roughly in 1/3 of patients) [36], supplemental oxygen should be administered during sleep to reach 90% saturations without frequent periods of >95% (to avoid hyperoxia-induced hypercapnia). When bi-level PAP is the chosen modality, some patients can be later safely switched to CPAP alone if they improve clinically. Ultimately weight loss is the definitive treatment. Since patient adherence is critical [36], great care should be taken to titrate therapies carefully and to customize treatment. Most important, noninvasive positive pressure therapies are a bridge to prevent worsening cardiopulmonary failure until patients lose weight; so clinicians must work tirelessly to help these patients lose the weight that is life threatening. With the current available data, noninvasive positive pressure therapies should never supplant endotracheal intubation and PPV for ACHRF if there are absolute indications (e.g., airway incompetence with aspiration, shock, profound excess work of breathing or tachypnea >35 breaths per minute with impending respiratory arrest) for securing the airway. Future studies may help to determine whether there are subsets of patients with ACHRF who benefit from positive pressure therapies, which should be used cautiously for such patients until such data are available.

## Figures and Tables

**Table 1:** Studies reporting clinical impact of positive pressure therapies on chronic hypercapnic respiratory failure.

Study	*n*/design	Mean BMI kg/m^2^	Intervention(s) (mean values in cmH_2_O)	Measurements when	Gas exchange	Symptoms	Sleep
Mokhlesi et al. [36]	75/Retro	51	CPAP = 14 Bilevel PAP = 18/13*	Median = 84 d	↓PCO_2_ 54 to 49 mmHg; *α* to adherence hours		

Piper et al. [37]	36/PR	53	CPAP = 14** Bilevel PAP = 16/10*	3 months	↓PCO_2_ 5.8 v. 6.9 mmHg	↓ Sleepiness both groups; ↑QOL > on bi-level PAP	Bilevel PAP better sleep quality

Hida et al. [38]	26/PNR	36	CPAP	3–6 months		↓ Sleepiness ↑QOL	

Banerjee et al. [39]	23/PNR	59	CPAP = 14	“later date”	↓TST < 80 and 90%		↓AHI, ↑REM

Berger et al. [23]	23/Retro	56	CPAP = 13 Bilevel PAP 18/8	Average of 14 months	↓PCO_2_ 55 to 45 mmHg		

Storre et al. [47]	10/Prospective	42	Bilevel PAP 15/6 AVAPS 16/5	6 weeks	↓PCO_2_ 47 to 42 in AVAPS and 46 in Bi-level PAP	↓Anxiety, ↑social functioning	↓Arousals, ↑REM, ↓RDI, ↓AHI

Pérez De Llano et al. [40]	54/Retro	44	Initial bilevel PAP settings 10/6	Mean of 50 months	↓PCO_2_ 61 to 44	↓Dyspnea	↓ESS 16 to 6

Masa et al. [43]	22/Prospective	41	NIMV***	4 months to a year	↓PCO_2_ 58 to 45	↓Headaches ↓Dyspnea ↓Edema	↓Sleepiness ↓Morning drowsiness

Pérez De Llano et al. [40]	24/Prospective	44	Bilevel PAP titration CPAP 10	24 hrs	↓PCO_2_ 58 to 44 in Bi-level PAP ↓PCO_2_ 60 to 42 in CPAP		↓ESS

Priou et al. [44]	130/Retro	44	Bilevel PAP IPAP 14 to 28 EPAP 6 to 14	6 months	↓PCO_2_ 56 to 45		↓ESS 10 to 4 ↓AHI 87 to 13

Murphy et al. [48]	50/PR	51	Bilevel PAP 25/10 AVAPS: volume = 657 mL EPAP = 9	3 months	↓PCO_2_ 51 to 47 in Bi-level PAP ↓PCO_2_ 53 to 48 in AVAPS		No between-group differences; intragroup comparisons not reported

Borel et al. [49]	35/PR	40	Bilevel PAP (versus counseling)	1 month	>↓PCO_2_ 48 to 43 in Bi-level PAP		↑REM 17 to 26% ↓AHI 40 to 6 In bi-level PAP

*Inspiratory pressure/expiratory pressure; **4 patients in CPAP group failed long term, requiring bilevel PAP, ***NIMV: volume cycled device or bilevel PAP device, PR: prospective randomized; PNR: prospective nonrandomized; Retro: retrospective; TST: total sleep time; QOL: quality of life; AHI: apnea-hypopnea index; REM: rapid eye movements; RDI: respiratory disturbance index; ESS: Epworth sleepiness score.
